# Shiga toxin delivery in extracellular vesicles induces high mortality and severe kidney injury in a Gb3-dependent manner

**DOI:** 10.1371/journal.ppat.1014421

**Published:** 2026-07-07

**Authors:** Markus Wendler, Alexandra Gerogianni, Johan Rebetz, Lazaro Hiram Betancourt Nunez, Patrik Önnerfjord, Ida Arvidsson, Diana Karpman

**Affiliations:** 1 Department of Pediatrics, Clinical Sciences Lund, Lund University, Lund, Sweden; 2 Department of Translational Medicine, Lund University, Lund, Sweden; 3 Mass Spectrometry, Clinical Sciences Lund, Lund University, Lund, Sweden; Tufts University School of Medicine, Boston, UNITED STATES OF AMERICA

## Abstract

*Escherichia coli* O157:H7 is a non-invasive Shiga toxin (Stx)-producing pathogen that causes gastroenteritis and hemolytic uremic syndrome characterized by acute kidney injury. Stx binding to cells induces release of extracellular vesicles (EVs) containing the toxin. This study aimed to demonstrate the lethal effects of toxin-positive vesicles, and the importance of the Stx receptor, globotriaosylceramide (Gb3), for these effects. HeLa cells were stimulated with Stx2 to induce vesicle release. EVs < 450 nm were isolated and cytotoxicity confirmed. BALB/c mice were injected intravenously with Stx2 126 ng/kg, the same toxin concentration in Stx2-EVs or EVs without toxin. Mortality was significantly increased in mice challenged with Stx2-EVs (n = 8/8) compared to mice challenged with free Stx2 (n = 4/7). Urea levels were high, and kidney tubulointerstitial pathology and fibrinogen staining prominent in mice challenged with Stx2-EVs. Mice challenged with EVs alone (n = 7) remained unaffected. C57BL/6 mice and Gb3-negative littermates were injected with Stx2-EVs at toxin concentrations of 126 or 200 ng/kg. Gb3-negative mice (n = 9) injected with Stx2-EVs at both toxin concentrations were totally protected whereas wild-type mice developed disease, n = 2/4 at the lower and 4/5 at the higher toxin concentration. These mice remained unaffected by the same concentrations of free toxin (n = 10) demonstrating that toxin delivered in vesicles was lethal. This study shows that Stx2 delivered systemically within EVs is lethal, causing severe kidney damage, and the Gb3 toxin receptor is crucial for disease induction by circulating Stx2-EVs.

## Author summary

Hemolytic uremic syndrome (HUS) is a severe complication of gastrointestinal infection with enterohemorrhagic *E. coli* (EHEC) that produce Shiga toxin and is the most common cause of acute kidney injury in young children in the Western world. Shiga toxin is divided into two main subgroups, Shiga toxin 1 and 2, and Shiga toxin 2 is more strongly associated with severe disease. EHEC strains do not cause bacteremia. Shiga toxin released from EHEC in the gut binds to blood cells and is released within extracellular vesicles. In this study, utilizing a mouse model of Shiga toxin-induced kidney injury, we compared the cytotoxic effects of Shiga toxin 2, delivered in extracellular vesicles or in free form, regarding kidney injury and mortality. Furthermore, the effect of toxin systemically delivered in extracellular vesicles was studied in mice that lack the Shiga toxin receptor globotriaosylceramide (Gb3) to determine if in vivo cytotoxicity induced by delivery in extracellular vesicles was Gb3-dependent. The study showed that toxin delivered in extracellular vesicles induced significantly more kidney injury and mortality and the effect was entirely Gb3-dependent, as Gb3-negative mice were totally protected from the effects of Shiga toxin-positive extracellular vesicles. The study provides a major step in understanding the mechanism of EHEC-associated kidney disease and mortality and demonstrates that the Gb3 receptor is a suitable therapeutic target for future studies.

## Introduction

Enterohemorrhagic *Escherichia coli* (EHEC) is a pathogen that colonizes the large intestine, causing diarrhea and hemorrhagic colitis. In severe cases, EHEC infection can progress to hemolytic uremic syndrome (HUS), a life-threatening condition characterized by acute kidney injury, thrombocytopenia, and microangiopathic hemolytic anemia [1] with no specific treatment available except supportive management [2]. EHEC, a subgroup of Shiga toxin (Stx)-producing *E. coli*, is a non-invasive pathogen that can produce Stx1 and/or Stx2 [3,4]. Stx is a potent virulence factor that gains access to the bloodstream [5,6] and Stx2 is more strongly associated with a severe clinical outcome [7]. Stx consists of a catalytic A-subunit and a pentameric B-subunit with a combined molecular weight of ~72 kDa. The A subunit functions as an RNA N-glycosidase, removing a specific adenine from 28S ribosomal RNA, thereby disrupting eukaryotic protein synthesis [8,9]. The B subunit binds to the glycosphingolipid globotriaosylceramide (Gb3) receptor present on blood cells such as erythrocytes, platelets and monocytes, as well as other cells including endothelial and epithelial cells [10–14].

Once bound to cells, the cells will release extracellular vesicles (EVs) that contain Stx2, a phenomenon our group and others previously demonstrated in EHEC-infected HUS patients [5,15] and in a murine model of EHEC infection [5]. Toxin-positive vesicles are released from blood cells, HeLa cells and other cells [5,14,16] and the toxin is bound to the Gb3 receptor on vesicles [14]. Stx2 associated with extracellular vesicles retains its toxicity [5]. Circulating Stx2-positive EVs undergo endocytosis by recipient cells whereby Stx2 is subsequently released from the EVs, eliciting a cytotoxic effect [5]. While recipient cells can internalize Stx2-positive EVs independently of Gb3 expression, cytotoxicity *in vitro* requires the presence of Gb3 in the recipient cell [16]. This aspect has not been addressed in animal models. *In vivo* experiments have shown that Stx-positive blood cell-derived EVs reach the kidney of mice orally infected with EHEC thereby causing kidney injury [5] and Stx2-positive HeLa cell derived-EVs injected intravenously into ICR mice caused lethality comparable to free Stx2 when using a low toxin concentration [17].

As EVs are involved in the transport of Stx2 to target organs, and are dependent on Gb3 for induction of cytotoxicity in the recipient cell *in vitro* [5,14], we aimed to investigate disease severity induced by Stx2-positive EVs in a murine model and the importance of Gb3 for development of disease. To address this, we utilized HeLa cell-derived Stx2-positive EVs and evaluated their toxicity *in vitro* and *in vivo* as well as the effect on kidney function and pathology. Gb3 synthase knockout mice were used to examine the importance of Gb3 for disease progression and survival outcome.

## Methods

### Ethics statement

All animal experiments were approved by the regional Animal Ethics Committee (approval 17452-20) and performed in accordance with regulations of the Swedish Board of Agriculture and the European Directive on the protection of animals used for scientific purposes. The number of mice was chosen to allow statistical comparison while complying with the 3R directives. All mandatory laboratory health and safety procedures were complied with, in the course of conducting in vitro and in vivo experimental work.

### Cell culture

HeLa cells (a kind gift from L. Johannes, Institute Curie, Paris, France) were maintained in Dulbecco’s Modified Eagle Medium (DMEM, HyClone Laboratories, Logan, UT) supplemented with 10% fetal calf serum (Gibco, Waltham, MA) and 1% penicillin-streptomycin (Gibco). Cells were cultured under standard conditions at 37°C in a humidified incubator with 5% CO₂.

### Shiga toxin 2

Shiga toxin 2 (Stx2) was obtained from the Tufts University Genomics Core at Tufts Medical School, Boston, MA. Limulus Amebocyte Lysate assay (Thermo Fisher Scientific, Waltham, MA) was performed detecting a lipopolysaccharide (LPS) concentration of 69 ng/mg of Stx2.

### Shiga toxin 2 stimulation of HeLa cells and extracellular vesicle purification

HeLa cells between 80–100% confluency in T175 cell culture flasks (Thermo Fisher Scientific) were stimulated with Stx2 10 ng/mL or phosphate-buffered saline (PBS) in DMEM supplemented with 1% exosome-depleted fetal bovine serum (Gibco) for 24 h on a shaker at 20 rpm at 37°C. Following stimulation, the supernatant was collected, the cells were washed once with PBS (HyClone Laboratories) supplemented with 25 mM HEPES (Gibco) and the wash combined with the supernatant. The pooled suspension of supernatant and wash was centrifuged at 300 × g for 10 min to remove cellular debris. The resulting supernatant was filtered through a 450 nm membrane filter (Pall Corporation, Port Washington, NY) to eliminate larger particles and isolate EVs. The filtrate was transferred to a 100 kDa molecular weight cutoff centrifugal filter (Sigma Aldrich, Burlington, MA) and centrifuged at 3,200 × g until approximately 90% of the suspension passed through the filter in order to remove free Stx2 and other substances less than 100 kDa. To remove residual contaminants, the retentate was washed twice with PBS-HEPES buffer (2 mL/filter) using the same centrifugal filter. The filter was inverted and placed into a Falcon tube, followed by centrifugation at 1,000 × g for 10 min to elute the EV-rich suspension. For quantification of Stx2 (described below) samples were diluted in PBS with 1% bovine serum albumin (BSA, Sigma Aldrich). Samples were kept at -80°C until used.

### Shiga toxin 2 quantification

White 96-well NUNC Maxisorp plates (Thermo Fisher Scientific) were coated with 100 µL/well of anti-Stx2 A subunit antibody 11F11 (BEI Resources, Manassas, VA) diluted to 1.3 µg/mL in 0.1 M carbonate buffer (pH 9.6) and incubated overnight at 4°C. Plates were washed thrice with PBS with 0.05% Tween-20 (PBS-T, Medicago AB, Uppsala, Sweden) 200 µL/well and blocked with PBS containing 1% BSA 100 µL/well for 1 h at RT. Toxin standards or EV samples with and without 0.5% saponin (Sigma Aldrich) were added 100 µL/well, and incubated overnight at RT. Plates were washed thrice with PBS-T and incubated with biotinylated (EZ-Link Sulfo-NHS-LC-Biotin, Thermo Fisher Scientific) anti-Stx2 A subunit antibody 11E10 (Santa Cruz Biotechnology, Dallas, TX) diluted to 0.52 µg/mL in blocking buffer at 100 µL/well for 1 h at RT. Plates were washed thrice with PBS-T and incubated with streptavidin poly-horseradish-peroxidase (HRP, Thermo Fisher Scientific) diluted to 50 ng/mL in blocking buffer at 100 µL/well for 1 h at RT. Plates were washed thrice and chemiluminescent detection was performed by SuperSignal ELISA Pico Chemiluminescent Substrate (Thermo Fisher Scientific) and measured by a GloMax Discover microplate reader (Promega Corporation, Madison, WI).

Analysis of EV samples showed a minimal difference in Stx2 levels with or without saponin-mediated membrane lysis. This suggested that most of the toxin was on the outside of the vesicles. Stx2 was exclusively found in EVs from Stx2-stimulated HeLa cells (Supporting information S1A), as expected.

### Vero cell assay

Vero cells were seeded into black 96-well plates with clear bottoms 10,000 cells/well (Corning Inc., Corning, NY) 48 h prior to the experiment. The Vero cells were washed twice with PBS 200 µL/well and incubated with either EVs released from Stx2-stimulated HeLa cells (termed Stx2-EVs) containing 10.7 ng/mL Stx2 (as determined by ELISA quantification described above), free Stx2 toxin (10.7 ng/mL), toxin-negative EVs from cells stimulated with PBS vehicle (termed neg-EVs), or PBS-HEPES, all diluted in RPMI medium (HyClone Laboratories) supplemented with 1% penicillin-streptomycin 100 µL/well and incubated with the cells for 24 h at 37°C. Neg-EVs concentration was equivalent to total protein content of Stx2-EVs measured using a Nanodrop spectrophotometer. Cell viability was assessed using the alamarBlue Cell Viability Reagent (Thermo Fisher Scientific), following the manufacturer’s protocol. Fluorescence signals were measured using the GloMax Discover microplate reader. Functionally, Stx2-EVs showed a cytotoxic effect comparable to free Stx2 in Vero cells. Stx2-EVs reduced viability by a median of 34%, whereas free Stx2 reduced viability by a median of 43% (Supporting information S1B showing data in relation to 100% viability defined by the vehicle and S1C showing the same data in arbitrary units of fluorescence).

### Extracellular vesicle size distribution

EV samples were analyzed for size distribution using nanoparticle tracking analysis (NTA). Samples were diluted 1:1000 in PBS-HEPES and loaded into a syringe pump. The EV suspensions were recorded under flow using a NanoSight LM10 instrument (Malvern Analytical). NTA of EVs samples showed a mode value of 103 nm (peak range 52–439 nm) for Stx2-positive EVs (Supporting information S2A) and 93 nm (peak range 93–484 nm) for Stx2-negative EVs (Supporting information S2B).

### Extracellular vesicle characterization

EV samples were lysed in 5X Passive Lysis Buffer (Promega Corporation, Madison, WI) and SDS, followed by incubation at RT for 15 min. The samples were subsequently boiled at 95°C for 5 min and loaded onto a 4–20% polyacrylamide gel (Bio-Rad Laboratories, Hercules, CA) for electrophoretic separation. Proteins were transferred onto a 0.2 µm PVDF membrane (Bio-Rad Laboratories) using a Trans-Blot Turbo Transfer System (Bio-Rad Laboratories). The membrane was blocked overnight at 4°C on a rocker in 1X Casein Solution (Vector Laboratories, Newark, CA). Membranes were incubated 1 h at RT with primary mouse antibodies against components of human EVs [18] at 1 µg/mL including TSG101, Alix (both from Invitrogen, Waltham, MA), CD63 (BD Biosciences, Franklin Lakes, NJ), CD81 and CD9 (both from Santa Cruz Biotechnology). Membranes underwent three 10 min washes in PBS-T and were incubated with a secondary antibody, goat anti-mouse HRP 1 µg/mL (Dako, Glostrup, Denmark), for 1 h at RT followed by three 10 min washes with PBS-T. Detection was performed using ECL Western Blotting Substrate (Thermo Fisher Scientific) incubated for 5 min. The chemiluminescent signals were visualized using a ChemiDoc Imaging System (Bio-Rad Laboratories). Both Stx2-positive and Stx2-negative EVs isolated from HeLa cell supernatants contained the EV markers TSG101 and CD63, and showed no detection of apolipoprotein A-I and B-100, suggesting the presence of EVs and lack of lipoprotein contamination (Supporting information S3A–S3D, for full blots see S9, S10).

### BALB/c mice injected with Stx2-EVs

BALB/c wild-type mice were bred and maintained at the animal facilities at Lund University under standard laboratory conditions. Both male and female mice, aged 8–12 weeks, were included in the study. Mice were injected intravenously into the tail vein with Stx2-EVs, free Stx2, neg-EVs, or PBS-HEPES as vehicle control. The concentration of Stx2 was 126 ng/kg for both Stx2-EVs and free Stx2, while neg-EVs were administered based on an equivalent total protein content. Mice were weighed daily and monitored two-three times daily, for weight loss, and clinical signs of illness such as ruffled fur, decreased activity, hunched posture, squinting, or neurological defects and given a clinical score of 0 (none), 1 (mild), 2 (moderate), 3 (severe), or 4 (death) presented in Supporting information S1 Table. In accordance with the ethical permit humane endpoints for euthanasia by cervical dislocation were defined as a clinical score higher than 0 or weight loss of ≥20%. All remaining mice were euthanized at the conclusion of the experiment (7 days post-treatment). All injections and clinical score assessments were conducted in a blinded manner. Blood samples were collected from tail snips into microvette tubes coated with EDTA (Sarstedt, Nümbrecht, Germany) for immediate blood count analysis using an XN-350 hematology analyzer (Sysmex, Kobe, Japan). Additional blood samples were obtained via heart puncture under isoflurane anesthesia before cervical dislocation, using syringes prefilled with 100 µL 0.1 M EDTA. Plasma was obtained by sequential centrifugation at 1500 × g for 15 min and 13,000 × g for 3 min. Plasma samples were stored at -80°C until analyzed. Kidneys were removed, fixed in 4% PFA (Histolab Products AB, Askim, Sweden) and embedded in paraffin. Tissues were sectioned at a thickness of 4 µm and mounted on glass slides for histological evaluation.

### C57BL/6 Gb3 synthase knock-out mice injected with Stx2-EVs

C57BL/6 wild-type and Gb3-negative (A4galt^tm1.1Poru^, a knock-out of Gb3 synthase) litter-mate mice, from R Sandhoff, Lipid Pathobiochemistry Group, German Cancer Research Center, Heidelberg, Germany [19], were bred at the animal facilities of Lund University. Genotyping of all Gb3-negative mice was performed by PCR to confirm the deletion, as described [20]. Both male and female mice, aged 8–12 weeks, were used in the study. Mice were injected intravenously with Stx2-EVs as above, free Stx2, neg-EVs, or PBS-HEPES vehicle and monitored as described above in a blinded manner. The concentration of Stx2 was 126 ng/kg or 200 ng/kg for Stx2-EVs, and 126 ng/kg, 200 ng/kg, or 500 ng/kg for free Stx2 injections. The highest concentration of free Stx2 was only given to wild-type mice.

### Blood urea nitrogen and creatinine levels in murine plasma

Blood urea nitrogen (BUN) and creatinine levels were measured in EDTA-plasma samples using the QuantiChrom Urea Assay kit or Creatinine Assay kit, respectively (BioAssay Systems, Hayward, CA), according to the manufacturer’s protocol. Each sample was analyzed in duplicate, and absorbance was measured using the GloMax Discovery microplate reader.

### Kidney histology and fibrinogen staining

Kidney tissue sections were deparaffinized and stained with hematoxylin-eosin to assess histopathological changes. Tissues were visualized using a Nikon Eclipse Ti-E microscope equipped with a Nikon color camera, and images were captured using NIS Elements AR software (v.5.11.01; Nikon Instruments Inc., Tokyo, Japan). Histopathological assessments of glomeruli and tubular interstitium in the entire section were performed. In glomeruli, thrombi and mesangial proliferation were assessed, in tubuli and interstitium tubular epithelial cell desquamation as well as interstitial edema were assessed and scored as 0 (absent), 1 (mild), 2 (moderate) and 3 (severe). Assessments were conducted in a blinded manner.

For fibrinogen and fibrin labelling, kidney sections were blocked with 5% BSA for 1 h at RT and incubated overnight at 4°C with rabbit anti-fibrinogen antibody 1 µg/mL (A0080, Dako, reacts with fibrinogen and with fibrin) in 1% BSA, or with control antibody rabbit IgG 1 µg/mL (Dako). Sections were washed thrice and incubated with goat anti-rabbit Alexa 488 5 µg/mL (Thermo Fisher Scientific) for 1 h at RT. Fluorescence intensity within each glomerulus was scored on a scale of 0–3. The score for each glomerulus was multiplied by the number of glomeruli exhibiting that score across the entire kidney section. The total fluorescence score was then divided by the total number of glomeruli in the section to calculate the mean fluorescence intensity per glomerulus. Assessments were conducted in a blinded manner.

### EV content characterization by mass spectrometry

EV samples were lysed in RIPA buffer (Sigma Aldrich) for 10 min, followed by ultrasonic disruption using a Bioruptor Plus (Diagenode) for 30 cycles (15 sec on, 15 sec off). Reduction, alkylation, ethanol precipitation and trypsin digestion was performed as previously described [21]. Peptide concentration was determined using a Nanodrop, and 500 ng were loaded onto disposable Evotip C18 trap columns (Evosep Biosystems) according to the manufacturer’s instructions.

Evosep One LC system (Evosep Biosystems) was used for separation of peptides with nanoflow reversed-phased chromatography with the 30 samples per day method (30 SPD, vendor standard settings) using a 15 cm × 150 µm Evosep column packed with 1.5 µm ReproSil-Pur C18- AQ particles. Samples were analyzed using a timsTOF Pro ion mobility mass spectrometer (Bruker Daltonics, Billerica, Massachusetts). Mass spectrometry (MS) data was acquired using data-independent acquisition serial fragmentation (DIAPASEF) method. The accumulation and ramp times were set to 100 ms. The collision energy was ramped linearly as a function of the mobility from 59 eV at 1/K0 = 1.5 Vs cm-2–20 eV at 1/K0 = 0.6 Vs cm-2.

### Proteomics data analysis

The MS raw files were searched for identification in Spectronaut Pulsar (version 19.9, Biognosys AG) using a combined reference proteome database from human (UP000005640), *E. coli* (UP000000625) and the Stx sequences (I6Y2S6 and I6Z6P8). For protein identification a false discovery rate <0.01 was used both at the protein and peptide level. Default settings (BGS factory settings) were used for the search with additional modifications: cysteine carbamidomethylation as a fixed modification, and N-terminal acetylation and methionine oxidation as variable modifications. At least one proteotypic peptide was required for protein identification. The precursor quantification was performed at MS2 level using only proteotypic and MaxLFQ for quantification. Cross-run normalization was performed using global normalization on median peptide intensity. Differentially abundant proteins were obtained using a paired t-test on log ratios (n = 3, different EV preparations).

To investigate enriched protein pathways and interactions for the identified proteins the g:Profiler search tool was employed [22]. A peptide abundance ratio cutoff of 2 and 0.5 for selection of relevant protein differences between the Stx2-EV and neg-EV samples was used with g:SCS multiple testing correction method applying a significance threshold of 0.05.

### Statistics

Statistical analysis was performed using R: A Language and Environment for Statistical Computing version 4.4.1 (R Foundation for Statistical Computing, Vienna, Austria). Multiple group comparisons were performed Kruskal–Wallis test followed by Dunn’s test. Survival analysis was performed using the Kaplan-Meier estimation of survival distribution test with the log-rank test for differences between groups. A two sided P value <0.05 was considered significant.

## Results

### Stx2-EVs induce high mortality in BALB/c mice

**Fig 1:**
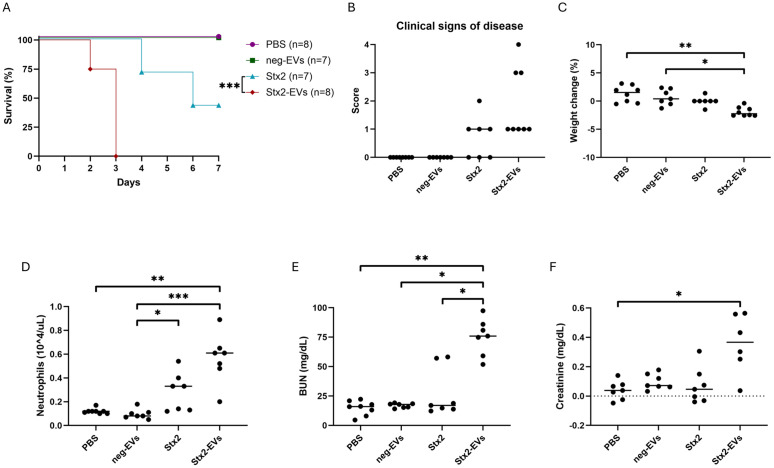
BALB/c mice challenged with Shiga toxin 2 (Stx2) and Stx2-EVs. BALB/c mice injected with PBS (n = 8, 4 male (M), 4 female (F)), neg-EVs (extracellular vesicles without Shiga toxin, n = 7, 3M,4F), free Shiga toxin 2 (Stx2) 126 ng/kg (n = 7, 4M,3F), or Stx2-EVs 126 ng/kg (n = 8, 5M,3F). All data obtained at the time of sacrifice. **A)** Survival curve of mice analyzed by the Kaplan-Meier estimation of survival distribution test. Statistical comparison of mice injected with Stx2-EVs compared to those injected with free toxin. **B)** Score of clinical disease in mice. Each dot represents one mouse, the bar represents the median. The score is the final score given to each mouse, as per S1 Table. **C)** Weight change on day 2. **D)** Neutrophil count in whole blood samples from mice taken at sacrifice. **E)** Blood urea nitrogen (BUN) levels in mice from plasma samples taken at sacrifice. A blood sample from one mouse injected with Stx2-EVs that developed symptom score 4 was lacking. **F)** Creatinine levels in plasma samples from BALB/c mice taken at sacrifice. Sufficient plasma samples were not available from two mice in the Stx2-EVs group. Multiple group comparisons were performed with Kruskal–Wallis test followed by Dunn’s test in panels C-F. *: P < 0.05, **: P < 0.01, ***: P < 0.001.

To assess the in vivo toxicity of Stx2-EVs, BALB/c mice were challenged with Stx2-EVs or free Stx2 and monitored for seven days. All mice receiving Stx2-EVs (n = 8) exhibited clinical signs of disease as evaluated by the clinical score in Supporting information S1 Table and were sacrificed by day 3 (Fig 1A, 1B). Mice were sacrificed solely due to clinical signs of disease, as weight loss did not exceed 20%. In contrast, mice injected with free Stx2 (n = 7) developed symptoms later (earliest on day 4), and 4/7 were sacrificed due to signs of disease (Fig 1A, 1B). Survival differences between mice injected with Stx2-EVs versus those injected with free Stx2 were statistically significant (P < 0.001, Fig 1A). Control groups receiving neg-EVs (n = 7) or PBS-HEPES (n = 8) remained asymptomatic throughout the experiment (Fig 1A, 1B). Mice challenged with Stx2-EVs exhibited significantly more weight loss (Fig 1C and Supporting information S4A–S4D) and elevated neutrophil counts (Fig 1D) compared to those challenged with neg-EVs or the PBS-HEPES controls. Additionally, blood urea nitrogen (BUN) and creatinine levels were significantly higher in the Stx2-EV group relative to all other groups (BUN) or the PBS group (creatinine, Fig 1E, 1F), indicating acute kidney injury.

### Stx2-EVs induced kidney pathology

**Fig 2:**
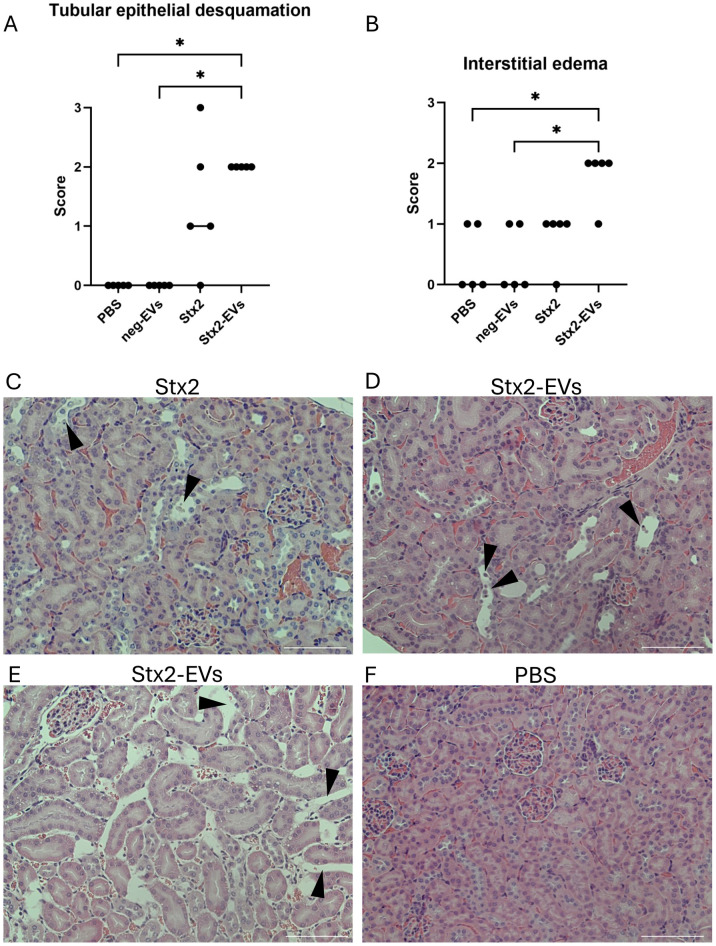
Kidney histology in BALB/c mice challenged with Stx2 and Stx2-EVs. **A)** Kidney section scores in BALB/c mice injected with PBS (n = 5, 3M, 2F), extracellular vesicles without Shiga toxin (neg-EVs, n = 5, 1M, 4F), Shiga toxin 2 (Stx2, n = 5, 2M, 3F), or extracellular vesicles with Stx2 (Stx2-EVs, n = 5, 4M, 1F) assessed for tubular epithelial desquamation. Scores were defined as 0 (absent), 1 (mild), 2 (moderate) and 3 (severe). **B)** Kidney section scores in BALB/c mice as in panel A assessed for intersitital widening suggestive of edema. **C)** Representative kidney section from mice injected with free Stx2. Arrowheads point to tubular epithelial desquamation. **D-E)** Representative kidney sections from mice injected with extracellular vesicles with Shiga toxin 2 (Stx2-EVs). **D)** Arrowheads indicate tubular epithelial desquamation. **E)** Arrowheads indicate interstitial widening suggestive of edema. **F)** A representative kidney section from a control mouse given PBS, showing normal mouse kidney histology. The bar corresponds to 100 μm. Multiple group comparisons were performed with Kruskal–Wallis test followed by Dunn’s test in panels A and B. *: P < 0.05.

Histopathological changes in kidney sections from mice showed increased tubulointerstitial kidney injury, as demonstrated by tubular cell desquamation (Fig 2A) and interstitial edema (Fig 2B), in mice challenged with Stx2-EVs compared to those challenged with PBS and neg-EVs. Tubular epithelial desquamation is presented in Fig 2C for a mouse injected with Stx2 and Fig 2D for a mouse injected with Stx2-EVs, and interstitial widening suggestive of edema is presented in Fig 2E for a mouse injected with Stx2-EVs. Control groups challenged with neg-EVs or PBS-HEPES exhibited minimal or no kidney pathology (Fig 2F). Pathological changes were not observed in glomeruli.

### Stx2-EVs induced fibrinogen/fibrin deposition in kidneys

**Fig 3:**
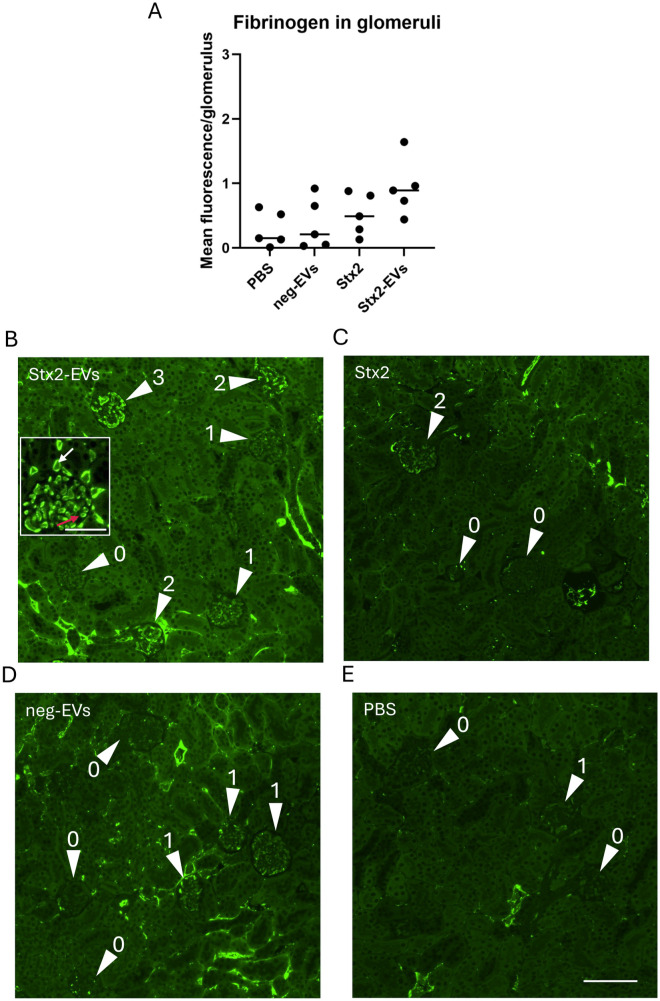
Fibrinogen and fibrin deposition in kidneys from BALB/c mice challenged with Stx2 and Stx2-EVs. **A)** Kidney sections from BALB/c mice injected with PBS (n = 5), extracellular vesicles without Shiga toxin (neg-EVs, n = 5), Shiga toxin 2 (Stx2, n = 5), or extracellular vesicles with Stx2 (Stx2-EVs, n = 5) and stained for fibrinogen and fibrin, the intensity was calculated and presented here as mean fluorescence/glomerulus. **B)** Fibrinogen/fibrin in a representative kidney section from a Stx2-EVs injected mouse. Numbers adjacent to the arrowheads pointing to glomeruli indicate the glomerular score (0-3). The inset shows labelling pointing to a glomerular capillary (red arrow) and a peritubular capillary (white arrow). The bar in the inset corresponds to 50 μm. **C)** Fibrinogen/fibrin in a representative kidney section from a Stx2-injected mouse. **D)** Fibrinogen/fibrin in a representative kidney section from a neg-EVs injected mouse. **E)** Fibrinogen/fibrin in a representative kidney section from a PBS-injected mouse. Multiple group comparisons were performed with Kruskal–Wallis test followed by Dunn’s test in panel A and did not achieve significance. The bar in panel E corresponds to 100 μm (same magnification in panels B-E).

Kidney sections from BALB/c mice were stained for fibrinogen and fibrin. Mice in the Stx2-EV group tended to have increased fibrinogen/fibrin deposition in glomeruli compared to other groups as summarized in Fig 3A. Stx2-EVs challenged mice exhibited an increased number of fibrinogen/fibrin-stained glomeruli and intensity of staining (Fig 3B) compared to the Stx2 (Fig 3C), neg-EVs (Fig 3D), and PBS-HEPES (Fig 3E) groups although this did not achieve statistical significance.

### Stx2-EVs only induce disease in Gb3-positive mice

**Fig 4:**
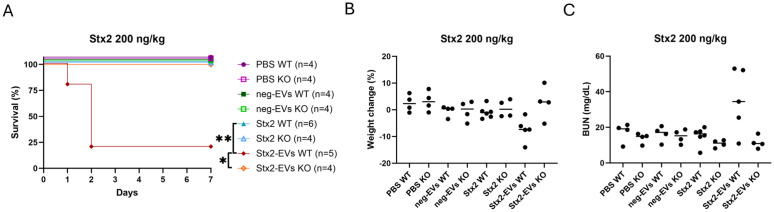
C57BL/6 wild-type and Gb3-negative mice challenged with Stx2 and Stx2-EVs. C57BL/6 wild-type (WT) and Gb3-negative (KO) mice injected with PBS (WT n = 4, 3M, 1F, KO n = 4, 1M, 3F), extracellular vesicles without Shiga toxin (neg-EVs, WT n = 4, 4M, KO n = 4, 2M, 2F), free Stx2 200 ng/kg (WT n = 6, 2M, 4F, KO n = 4, 2M, 2F), or extracellular vesicles with Stx2 (Stx2-EVs) 200 ng/kg (WT n = 5, 1M, 4F, KO n = 4, 1M, 3F). **A)** Survival curve of mice, 4/5 wild-type mice injected with Stx2-EVs developed symptom score 2 (as per S1 Table) and were sacrificed. **B)** Weight change from day 1 to day of sacrifice. Each dot represents one mouse, the bar represents the median. **C)** Blood urea nitrogen (BUN) levels in mice. Statistical comparisons of survival (panel A) in wild-type mice injected with Stx2-EVs compared to Gb3-negative mice injected with Stx-EVs, and compared to wild-type mice injected with free Stx2. Survival curve analyzed by the Kaplan-Meier estimation of survival distribution test. *: P < 0.05, **: P < 0.01. Statistical comparisons in panels B and C (multivariate analysis) were not significant.

To evaluate the role of the Gb3 receptor in Stx2-EV-mediated toxicity, C57BL/6 wild-type and Gb3-negative mice were challenged with Stx2-EVs, free Stx2, neg-EVs or PBS-HEPES and monitored for seven days. Wild-type mice injected with Stx2-EVs at a Stx2 concentration corresponding to 200 ng/kg, developed clinical signs of disease starting on day 1, and 4/5 mice were euthanized by day 2 (Fig 4A). These mice also developed more weight loss than the other groups (Fig 4B and S5A–S5D information), as well as higher levels of BUN (Fig 4C). Wild-type mice injected with Stx2-EVs at a concentration corresponding to 126 ng/kg exhibited symptoms on day 2 (2/4) and were sacrificed (S6A information). All mice in this group exhibited more weight loss compared to all other groups (S6B–S6D information) but comparable levels of BUN (S6E information). In contrast, all Gb3-negative mice, injected with Stx2-EVs at 200 ng/kg (n = 4) or 126 ng/kg (n = 5), remained asymptomatic throughout the entire experiment. There was a significant difference in the survival rate of Gb3-negative mice injected with Stx2-EVs (200 ng/kg) compared to wild-type mice (P < 0.05, Fig 4A).

Wild-type mice injected with free Stx2 200 and 126 ng/kg (n = 6 and n = 4, respectively) did not develop signs of disease and all these mice survived compared to wild-type mice injected with Stx2-EVs (Fig 4A and S6A information). When given Stx2 200 ng/kg survival differences were significant compared to mice given Stx2-EVs, P < 0.01, Fig 4A. As these mice seemed to be resistant to free toxin at 200 ng/kg, mice were injected with a higher concentration of Stx2 at 500 ng/kg. At this concentration wild-type mice exhibited clinical signs of disease by day 2, and 4/4 mice were euthanized on day 2 (S7 information).

### Mass spectrometry of HeLa cell-derived EVs

**Fig 5:**
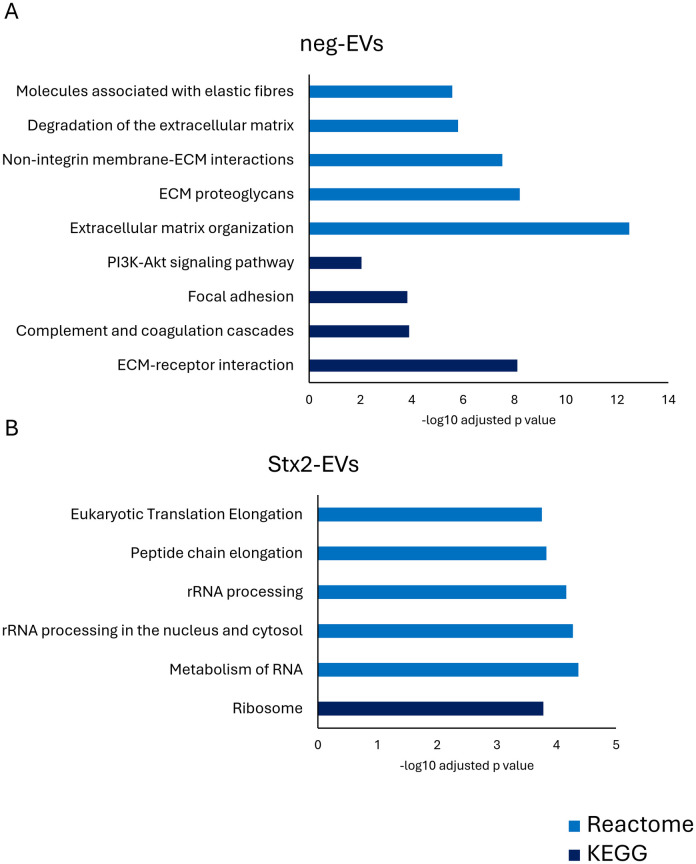
Mass spectrometry of extracellular vesicle samples. Search tool results of pathway enrichment analysis from the Reactome and KEGG database.The top 5 significant enriched pathways are annotated with exclusion of pathways specifically related to other infections (amebiasis, COVID-19 and toxoplasmosis). **A)** Enriched pathways in neg-EVs (extracellular vesicles without Shiga toxin). **B)** Enriched pathways in extracellular vesicles with Stx2 (Stx2-EVs).

To determine whether toxin-negative EVs differ in molecular cargo compared to toxin-positive EVs, beyond the presence of Stx2 itself, a comprehensive MS-based proteomic analysis was performed. As anticipated, Stx2 was exclusively detected in EVs isolated from Stx2-stimulated cells, confirming the selective incorporation of the toxin. The g:Profiler search tool found pathways annotated in the Reactome [23] and KEGG databases [24] that are associated with the complement and coagulation cascade and were higher in neg-EVs relative to Stx2-EVs (Fig 5A, 5B and S8A–S8B information). These findings suggest that, beyond the presence of Stx2, the Stx2-EVs did not incorporate other inflammatory EV cargo. The proteomics data have been deposited to the ProteomeXchange Consortium via the PRIDE [25] partner repository with dataset identifier PXD065687.

## Discussion

This study demonstrates that circulating Stx2-EVs induce severe kidney injury and mortality. Gb3-negative mice were totally protected from the effects of Stx2-EVs. Thus, the enhanced Stx2 potency is contingent upon the presence of the Gb3 receptor in the target cell with which the Stx2-EVs interact. Proteomic analysis of EVs revealed that there were few differences in the content of EVs, with and without the bacterial toxin, apart from the presence of Stx2, with some immune-related pathways being downregulated in Stx2-EVs, suggesting that the observed increase in toxicity related to Stx2-EVs is attributable to the presence of Stx2. These findings highlight the essential role of bacterial toxin delivery to target organs by EVs and the importance of the Gb3 receptor for mediating Stx2-EV toxicity.

Stx2 delivered by EVs was more potent, causing more lethality and kidney injury than free toxin even though free toxin and toxin associated with EVs exhibited comparable cytotoxicity in a Vero cell assay. One previous study using female ICR mice and a lower concentration of toxin in EVs showed that free toxin and Stx2 associated with EVs had a similar effect on survival and that toxin associated with EVs caused more kidney pathology [17]. In the current study we used BALB/c mice and a higher concentration of Stx2 with results showing a distinct difference between Stx2-EVs and free Stx2. BALB/c mice injected with Stx2-EVs had the highest levels of urea, neutrophil counts, increased tubular-interstitial pathology, and a tendency for increased fibrinogen and fibrin deposition, indicating that Stx2-EVs induced inflammation and injured the kidney, more than free toxin. This may reflect enhanced delivery or increased bioavailability of the toxin when associated with EVs, facilitating its transport to target tissues. Another possibility is that Stx2 is protected from the host response when sequestered within the EVs allowing it to evade the immune response. This would require the toxin to be localized within vesicles. In previous studies using Stx1 we found more toxin within HeLa cell-derived EVs than on the outside [14] but in the current study most of the Stx2 was present on the outside of vesicles. This could be due to variations in the freeze-thaw process and the method used for EV isolation or properties of the toxin itself, Stx1 versus Stx2, when used *in vitro*. However, it is important to note that in patient plasma samples most of the Stx2 is present within EVs [5], which most probably reflects the *in vivo* setting. Furthermore, using Stx1, we have shown that the toxin can undergo translocation thru the membrane [14] which suggests a fluidity in its localization.

The issue of Stx2 localization on the inside or outside of EVs could be of importance for designing treatment options. Stx2 on the outside of EVs could be targeted by specific antibodies, such as recently shown with moderately beneficial effects [26]. However, if most of the toxin is not exposed *in vivo* these treatments would not neutralize the toxin if they cannot gain access to the vesicle membrane. Thus, novel treatments should be capable of uptake in the cell and vesicle membrane.

Our group has previously shown that 3–4 days after intraperitoneal injection of Stx2, at a concentration of 285 ng/kg, to BALB/c mice, Stx2-positive platelet-derived EVs were detected in the circulation [5]. The concentration of toxin used in the present study was lower but presumably a similar distribution would occur in the circulation after intravenous injection. Injected Stx2 would bind to circulating cells exposing the Gb3 receptor and be taken up by the cells. Some of these cells, such as platelets, would release the toxin within EVs that would then circulate and reach the target organ, i.e., the kidney. Prepackaged Stx2 in EVs from HeLa cells may therefore cause kidney injury sooner than free toxin that will first bind to blood cells. This potential difference in the kinetics of Stx-containing EV formation could explain why mice injected with free toxin developed disease later but does not explain the difference in disease severity. The latter may be partially explained by loss of free toxin after intravenous injection as the toxin is removed by cells and only a fraction will be released in EVs containing toxin. The findings suggest that packaging of Stx2 in EVs is a very potent mechanism of bacterial toxin transfer.

Differences in the content of EVs containing Stx2 and those without toxin were analyzed by mass spectrometry. The purpose was to determine if bacterial or human factors, other than Stx2, could contribute to the toxic effects of the Stx2-positive EVs. Although this was not found to be the case the results showed that EVs lacking toxin were enriched for complement or coagulation cascades whereas EVs containing toxin were enriched for RNA and ribosomal pathways. Shiga toxin induces cytotoxicity by depurination of a specific adenine residue in 28S ribosomal RNA [8] and, thus, the upregulation of ribosomal pathways may be a cellular response to this cytotoxic pathway within HeLa cells. This aspect requires future study.

Several studies have addressed the importance of the Gb3 receptor for Stx2 toxicity *in vivo* [27–29], and even though Stx may bind to Gb4 [30], trafficking and toxicity *in vitro* and *in vivo* have been associated with binding to Gb3 [31–33]. Our group has demonstrated that Stx2, after binding to blood cells or HeLa cells, is released in EVs [5,14] and the latter vesicles possess the Gb3 receptor to which the toxin is bound [14]. Circulating Stx2-EVs are taken up by target organ cells in the kidney [5]. EV uptake by cells is, in general, not a Gb3-dependent process [34] and consequently, Stx2-EV uptake by cells is not Gb3-dependent [16]. We therefore investigated if Stx2 toxicity is solely mediated by the presence of the Gb3 receptor on the EVs, and found that the recipient cell must possess the Gb3 receptor for Stx2-EVs to induce cytotoxicity *in vitro* [16]. Here we show that the injurious effects of Stx2-EVs *in vivo* are Gb3-dependent as well. While wild-type mice succumbed to Stx2-EV injection the Gb3-negative litter-mate mice remained unaffected. This is ultimate evidence of the importance of Gb3 for the toxicity of Stx2-EVs and shows that any potential contribution from other virulence factors, including trace amounts of LPS, is negligible.

A potential limitation of the experimental design is that, in accordance with the approved ethical protocol, animals were euthanized upon the first signs of clinical disease. As the assessment of clinical symptoms is inherently subjective, measures were implemented to minimize observer bias. Specifically, clinical scoring was performed by the same investigator at all time points, and the investigator remained blinded to the treatment allocation throughout the study.

Patients with EHEC-HUS have been shown to have Stx in their kidneys, thereby explaining the kidney injury induced during EHEC infection [5,35]. Similarly, Stx2 injected intravenously in mice was detected in the kidney [29]. We have shown that Stx2 targets the kidney by circulating in blood cell-derived EVs that are taken up by kidney cells, demonstrated in a patient with HUS and in EHEC-infected mice [5]. In the current study we chose to use HeLa cell-derived EVs instead of blood cell-derived EVs. These were selected due to the well-characterized nature of the cell line, the ability to yield reproducible results and our previous report of the presence of Gb3 on EVs released from HeLa cells [14]. Eliglustat is an inhibitor of glucosylceramide synthase, approved for Gaucher disease. It has been tested *in vitro* by others and shown to reduce Gb3 and the effects of Shiga toxin on cells [36]. As Gb3 is crucial for the development of Shiga toxin-mediated disease future studies should address the effects of eliglustat in EHEC-associated disease. Targeting the Gb3 could be a novel therapeutic intervention that would neutralize the effects of toxin incorporated in EVs and thereby delivered to target organs.

This study provides insight into EHEC pathogenesis whereby Stx2 delivery in EVs is lethal in a mouse model that recapitulates Stx2-EV transfer to target organs. The findings highlight the essential role of the Gb3 receptor in mediating Stx2-EV toxicity demonstrating that Stx2-EVs induce severe kidney injury and mortality. The study therefore provides a major step in our understanding of the mechanism of EHEC-associated kidney disease and mortality and demonstrates that the Gb3 receptor is a suitable therapeutic target for future studies.

## Supporting information

S1 Fig Shiga toxin 2 associated with extracellular vesicles are cytotoxic in vitro. **A)** Shiga toxin 2 (Stx2) concentration of extracellular vesicles (EV) samples from HeLa cell culture stimulated with or without Stx2 compared to the respective batch of flowthrough (FT), flowthrough from first wash (W1), and second wash (W2). **B)** Cell viability assay of Vero cells treated with toxin-negative EVs (neg-EVs), free Stx2 10.7 ng/mL, or toxin-positive EVs (Stx2-EVs) 10.7 ng/mL compared to the control group treated with PBS defined as 100%. **C)** Cell viability assay showing arbitrary units of fluorescence in the Y axis corresponding to panel B. Multiple group comparisons were performed with Kruskal–Wallis test followed by Dunn’s test. *: P < 0.05, ***: P < 0.001.
(TIF)

S2 Fig Nanotracking analysis of extracellular vesicles. **A)** Shiga toxin 2-extracellular vesicle size, compilation of 5 runs. **B)** Toxin-negative extracellular vesicles (neg-EVs) compilation of 5 runs.
(TIF)

S3 Fig Immunoblot of extracellular vesicle samples. Lysed toxin-negative EVs (neg-EVs) and Shiga toxin positive EVs (Stx2-EVs) run on immunoblot with antibodies against components of human EVs or lipoprotein contaminants. **A)** Anti-TSG101 (Tumor Susceptibility Gene 101), ~ 46 kDa. **B)** Anti-CD63, ~ 53 kDa. **C)** Anti-ApoA (Apolipoprotein A1), ~ 28 kDa. **D)** Anti-ApoB (Apolipoprotein B), ~ 260–500 kDa. Uncropped full blots are presented in Supporting information S9-S10.
(TIF)

S1 Table Symptoms score used for clinical signs of disease. (TIF)

S4 Fig Weight changes in BALB/c mice challenged with Stx2 126 ng/kg and Stx2-EVs. Weight loss curve of BALB/c mice injected with **A)** PBS (n = 8, 4 male (M), 4 female (F)). **B)** Neg-EVs (extracellular vesicles without Shiga toxin, n = 7, 3M, 4F). **C)** Free Shiga toxin 2 (Stx2) 126 ng/kg (n = 7, 4M, 3F). **D)** Stx2-EVs 126 ng/kg (n = 8, 5M, 3F). Body weight in mice was measured once daily.
(TIF)

S5 Fig Weight change in C57BL/6 wild-type and Gb3-negative mice challenged with Stx2 200 ng/kg and Stx2-EVs. Weight loss curve of C57BL/6 wild-type (WT) and Gb3-negative (KO) mice injected with **A)** PBS (4 WT, 4 KO). **B)** Extracellular vesicles without Shiga toxin (neg-EVs, 4 WT, 4 KO). **C)** Free Stx2 200 ng/kg (6 WT, 4 KO). **D)** Stx2-EVs 200 ng/kg (5 WT, 4 KO). Each curve represents one mouse and each dot is one weight measurement.
(TIF)

S6 Fig C57BL/6 wild-type and Gb3-negative mice challenged with Stx2 126 ng/kg and Stx2-EVs. C57BL/6 wild-type (WT) and Gb3-negative (KO) mice injected with free Stx2 126 ng/kg (WT n = 4, 3M, 1F, KO n = 4, 2M, 2F), or extracellular vesicles with Stx2 (Stx2-EVs) 126 ng/kg (WT n = 4, 1M, 3F, KO n = 5, 4M, 1F). **A)** Survival curve of mice, 2/4 wild-type mice injected with Stx2-EVs developed clinical score 2 (as per S1 Table) and were sacrificed. Analysis by the Kaplan-Meier estimation of survival distribution test was not significant. **B)** Weight change from day 1 to day of sacrifice. **C)** Body weight change in mice measured once daily. Each curve represents one mouse. **E)** Blood urea nitrogen (BUN) levels in mice. One plasma sample was lacking from a mouse in the Stx2-EVs WT group. Each dot represents one mouse, the bar represents the median. Survival curve analyzed by the Kaplan-Meier estimation of survival distribution test in panel A and was not significant.
(TIF)

S7 Fig C57BL/6 wild-type mice challenged with Stx2 500 ng/kg. Survival curve of C57BL/6 wild-type (WT, n = 4, 3F, 1M) mice injected with 500 ng/kg of Shiga toxin 2 (Stx2). The 4 mice with clinical signs of disease developed clinical scores 3, 2, 2 and 1 as per S1 Table. This experiment aimed to demonstrate that the mice could respond to free Stx2 at a higher concentration and was terminated after 3 days.
(TIF)

S8 Fig Mass spectrometry of extracellular vesicle samples. The top 20 proteins with highest fold changes in **A)** neg-EVs. **B)** Stx2-EVs.
(TIF)

S9 Fig Uncropped immunoblot of extracellular vesicle samples shown in S3A and S3B. Lysed toxin-negative EVs (neg-EVs) and Shiga toxin positive EVs (Stx2-EVs) run on immunoblot with antibodies against components of human EVs. **A)** Anti-TSG101 (Tumor Susceptibility Gene 101), ~46 kDa. **B)** Anti-CD63, ~53 kDa.
(TIF)

S10 Fig Uncropped immunoblot of extracellular vesicle samples shown in S3C and S3D. Lysed toxin-negative EVs (neg-EVs) and Shiga toxin positive EVs (Stx2-EVs) run on immunoblot with antibodies against lipoprotein contaminants. **A)** Anti-ApoA (Apolipoprotein A1), ~28 kDa. **B)** Anti-ApoB (Apolipoprotein B), ~260–500 kDa.
(TIF)
