# Supplementary figures and images for: Shiga toxin delivery in extracellular vesicles induces high mortality and severe kidney injury in a Gb3-dependent manner
Source: PLoS Pathog. 2026 Jul 7;22(7):e1014421. doi: 10.1371/journal.ppat.1014421 (PMC13367898; doi:10.1371/journal.ppat.1014421)

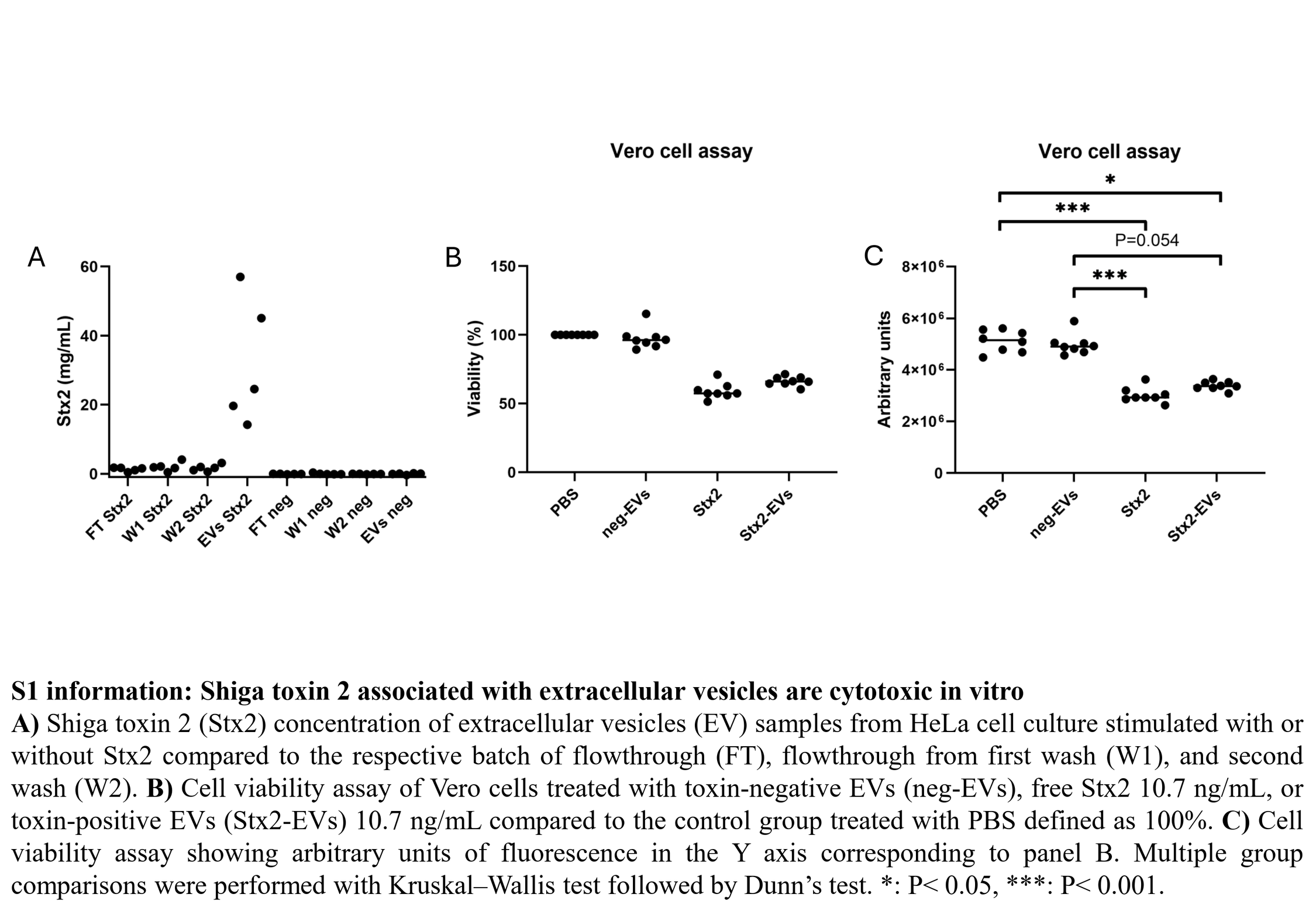

Supplement: S1 Fig — A) Shiga toxin 2 (Stx2) concentration of extracellular vesicles (EV) samples from HeLa cell culture stimulated with or without Stx2 compared to the respective batch of flowthrough (FT), flowthrough from first wash (W1), and second wash (W2). B) Cell viability assay of Vero cells treated with toxin-negative EVs (neg-EVs), free Stx2 10.7 ng/mL, or toxin-positive EVs (Stx2-EVs) 10.7 ng/mL compared to the control group treated with PBS defined as 100%. C) Cell viability assay showing arbitrary units of fluorescence in the Y axis corresponding to panel B. Multiple group comparisons were performed with Kruskal–Wallis test followed by Dunn’s test. *: P < 0.05, ***: P < 0.001. (TIF) [file ppat.1014421.s001.tif]

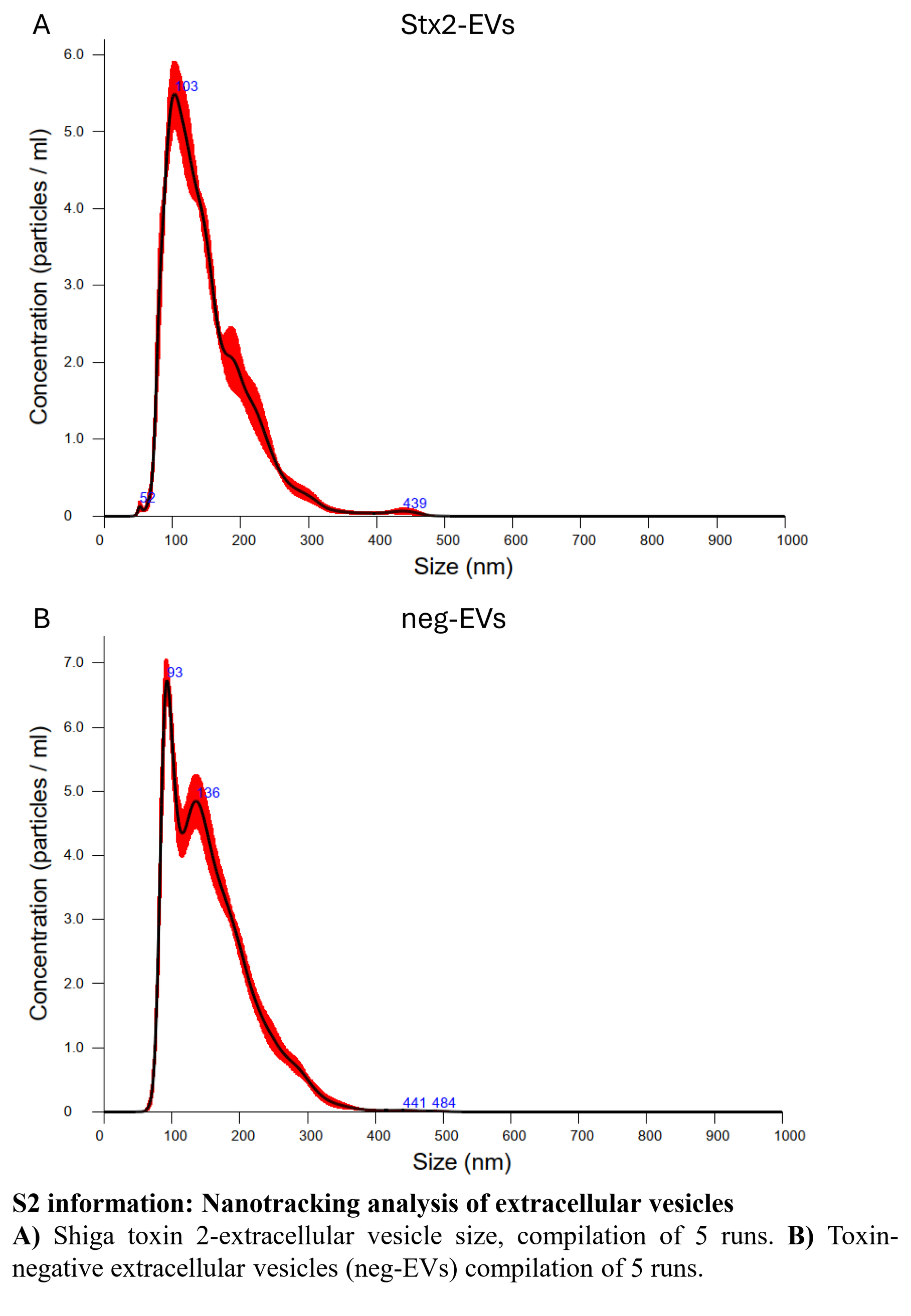

Supplement: S2 Fig — A) Shiga toxin 2-extracellular vesicle size, compilation of 5 runs. B) Toxin-negative extracellular vesicles (neg-EVs) compilation of 5 runs. (TIF) [file ppat.1014421.s002.tif]

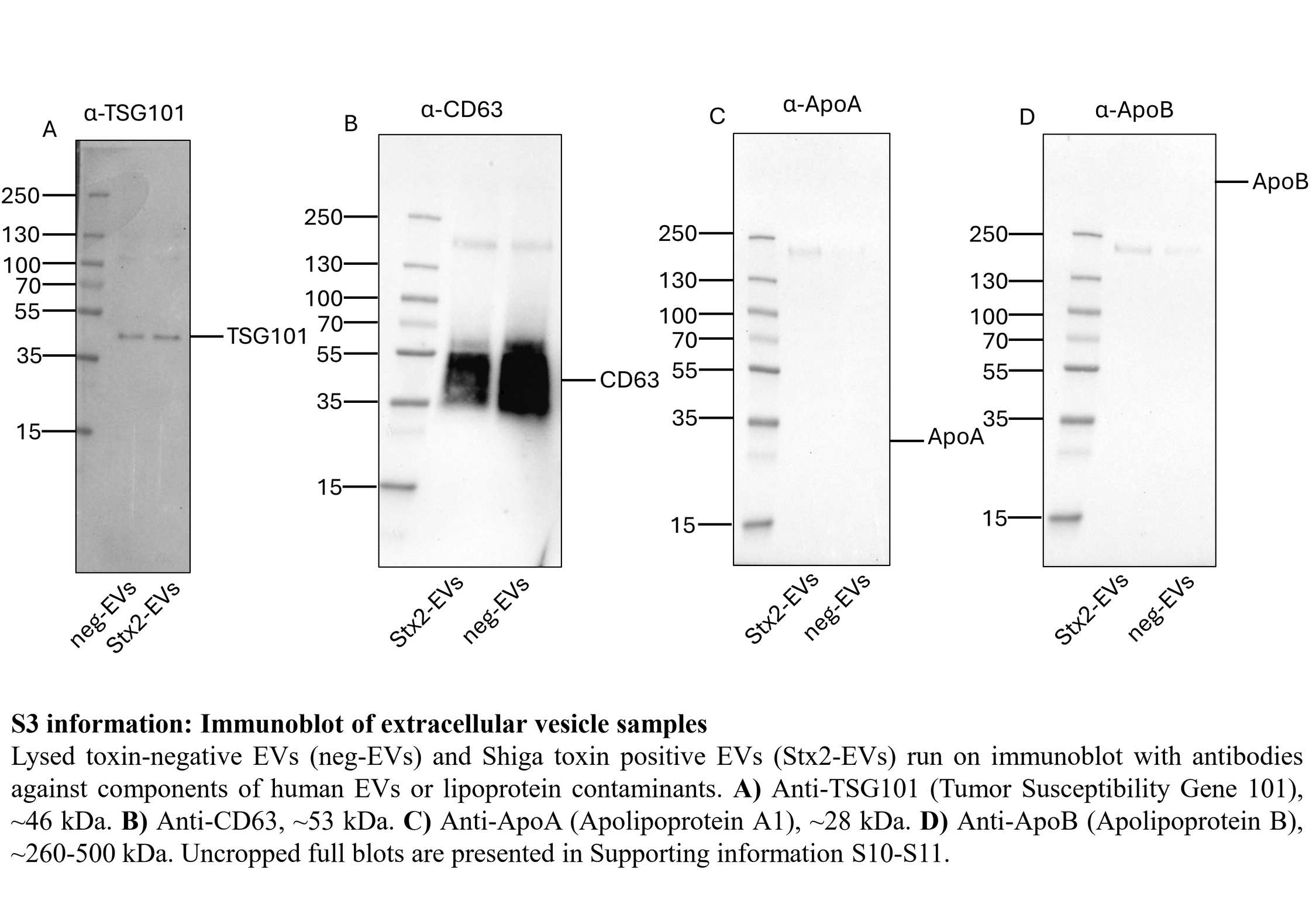

Supplement: S3 Fig — Lysed toxin-negative EVs (neg-EVs) and Shiga toxin positive EVs (Stx2-EVs) run on immunoblot with antibodies against components of human EVs or lipoprotein contaminants. A) Anti-TSG101 (Tumor Susceptibility Gene 101), ~ 46 kDa. B) Anti-CD63, ~ 53 kDa. C) Anti-ApoA (Apolipoprotein A1), ~ 28 kDa. D) Anti-ApoB (Apolipoprotein B), ~ 260–500 kDa. Uncropped full blots are presented in Supporting information S9-S10. (TIF) [file ppat.1014421.s003.tif]

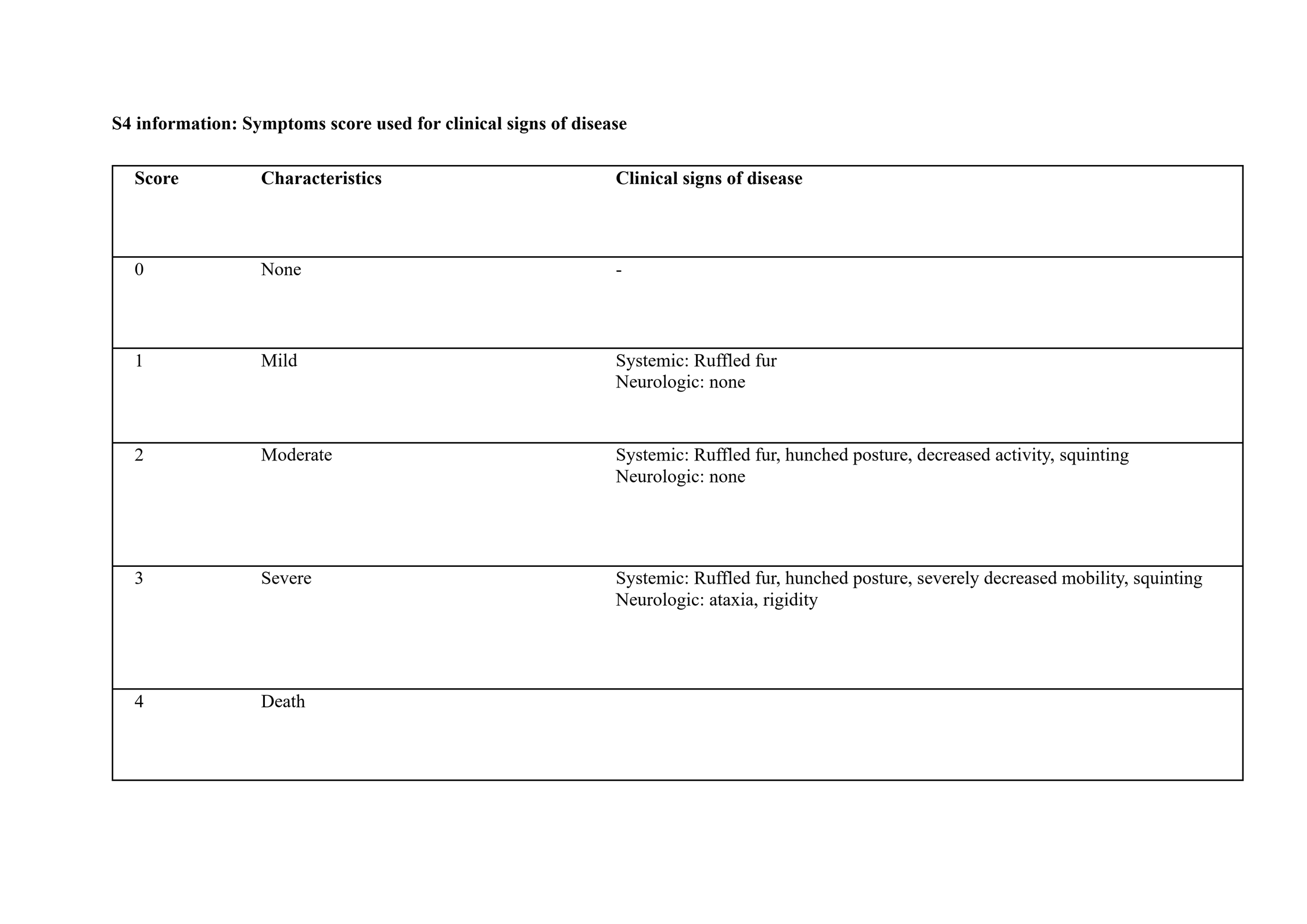

Supplement: S1 Table — (TIF) [file ppat.1014421.s004.tif]

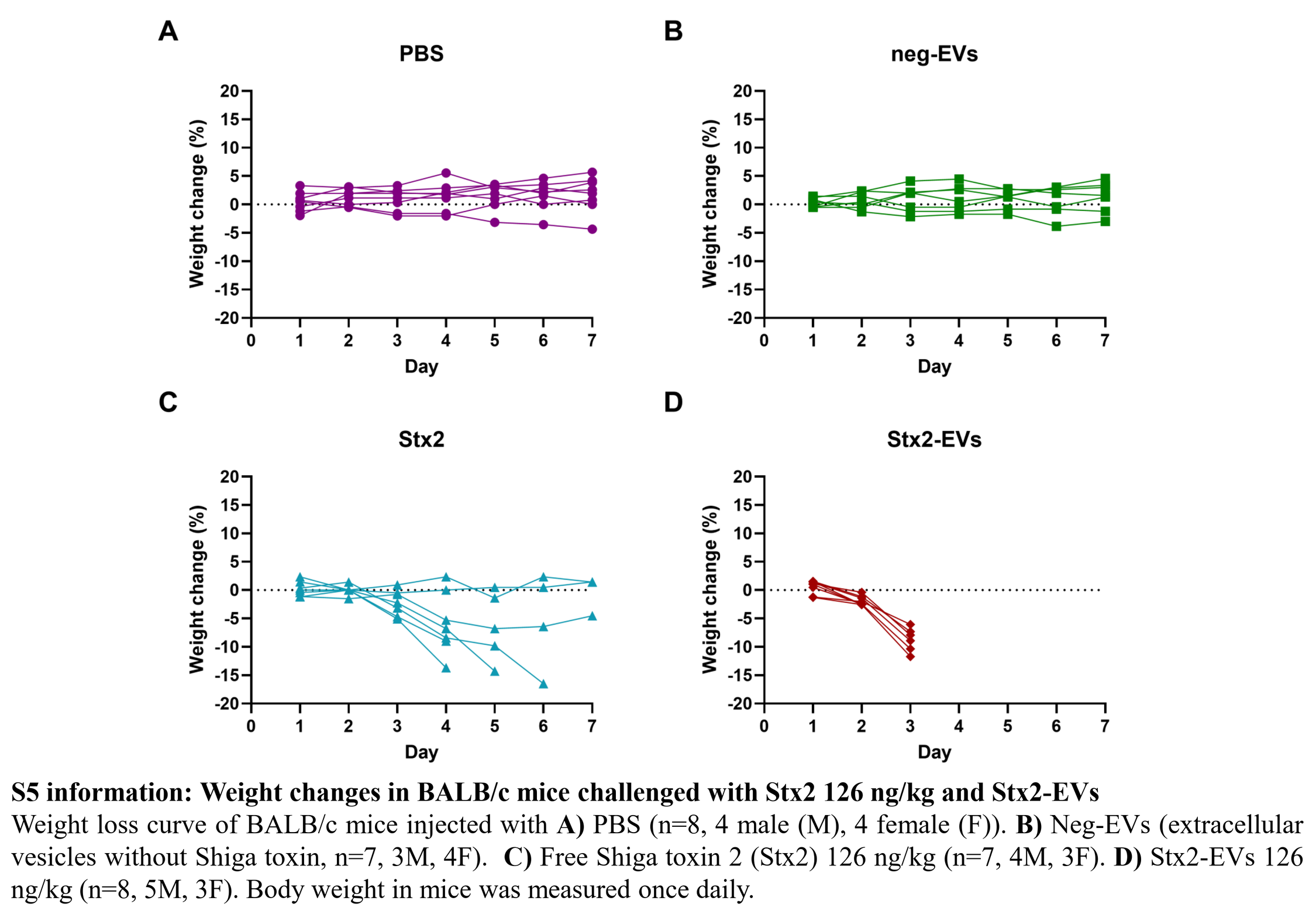

Supplement: S4 Fig — Weight loss curve of BALB/c mice injected with A) PBS (n = 8, 4 male (M), 4 female (F)). B) Neg-EVs (extracellular vesicles without Shiga toxin, n = 7, 3M, 4F). C) Free Shiga toxin 2 (Stx2) 126 ng/kg (n = 7, 4M, 3F). D) Stx2-EVs 126 ng/kg (n = 8, 5M, 3F). Body weight in mice was measured once daily. (TIF) [file ppat.1014421.s005.tif]

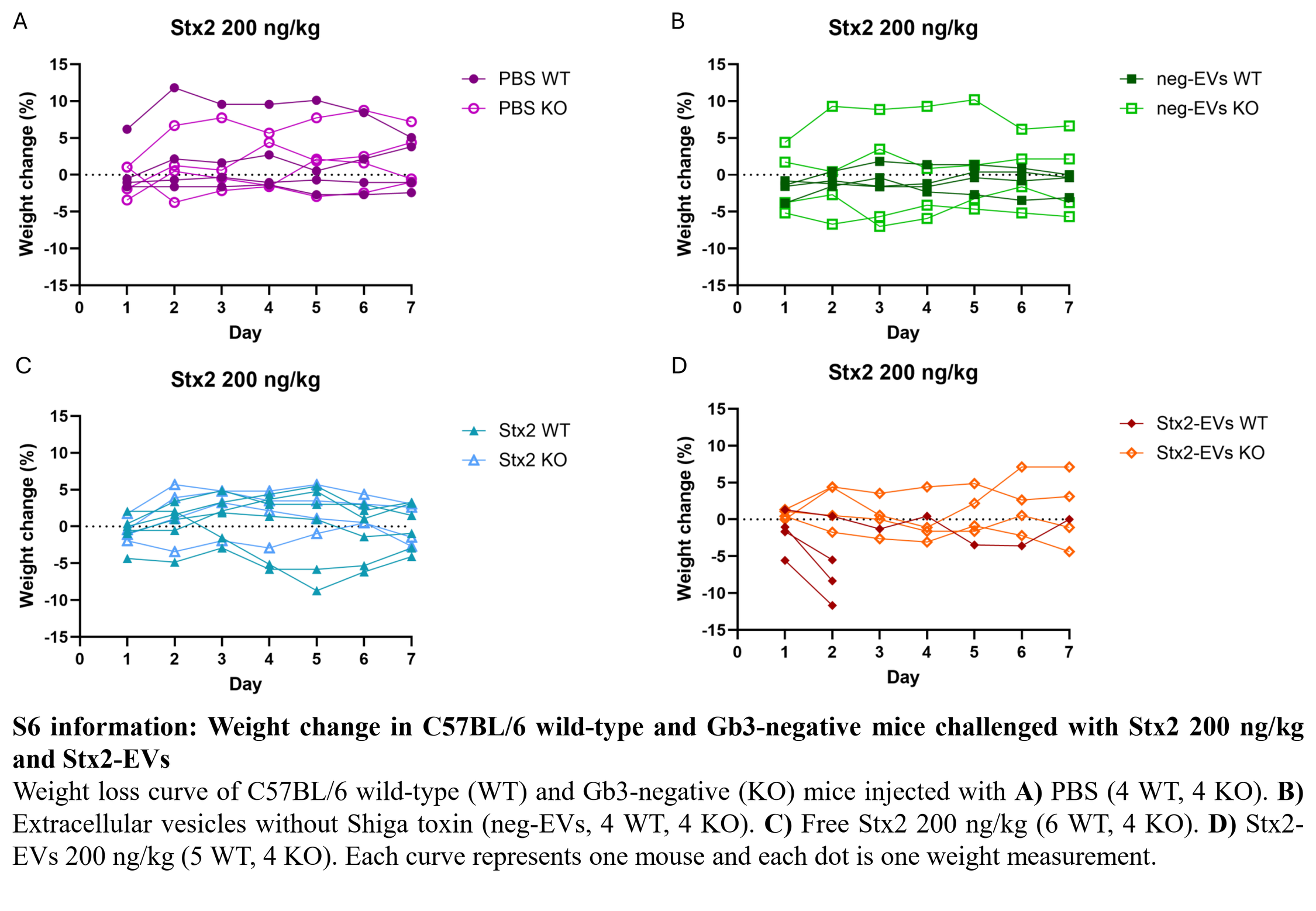

Supplement: S5 Fig — Weight loss curve of C57BL/6 wild-type (WT) and Gb3-negative (KO) mice injected with A) PBS (4 WT, 4 KO). B) Extracellular vesicles without Shiga toxin (neg-EVs, 4 WT, 4 KO). C) Free Stx2 200 ng/kg (6 WT, 4 KO). D) Stx2-EVs 200 ng/kg (5 WT, 4 KO). Each curve represents one mouse and each dot is one weight measurement. (TIF) [file ppat.1014421.s006.tif]

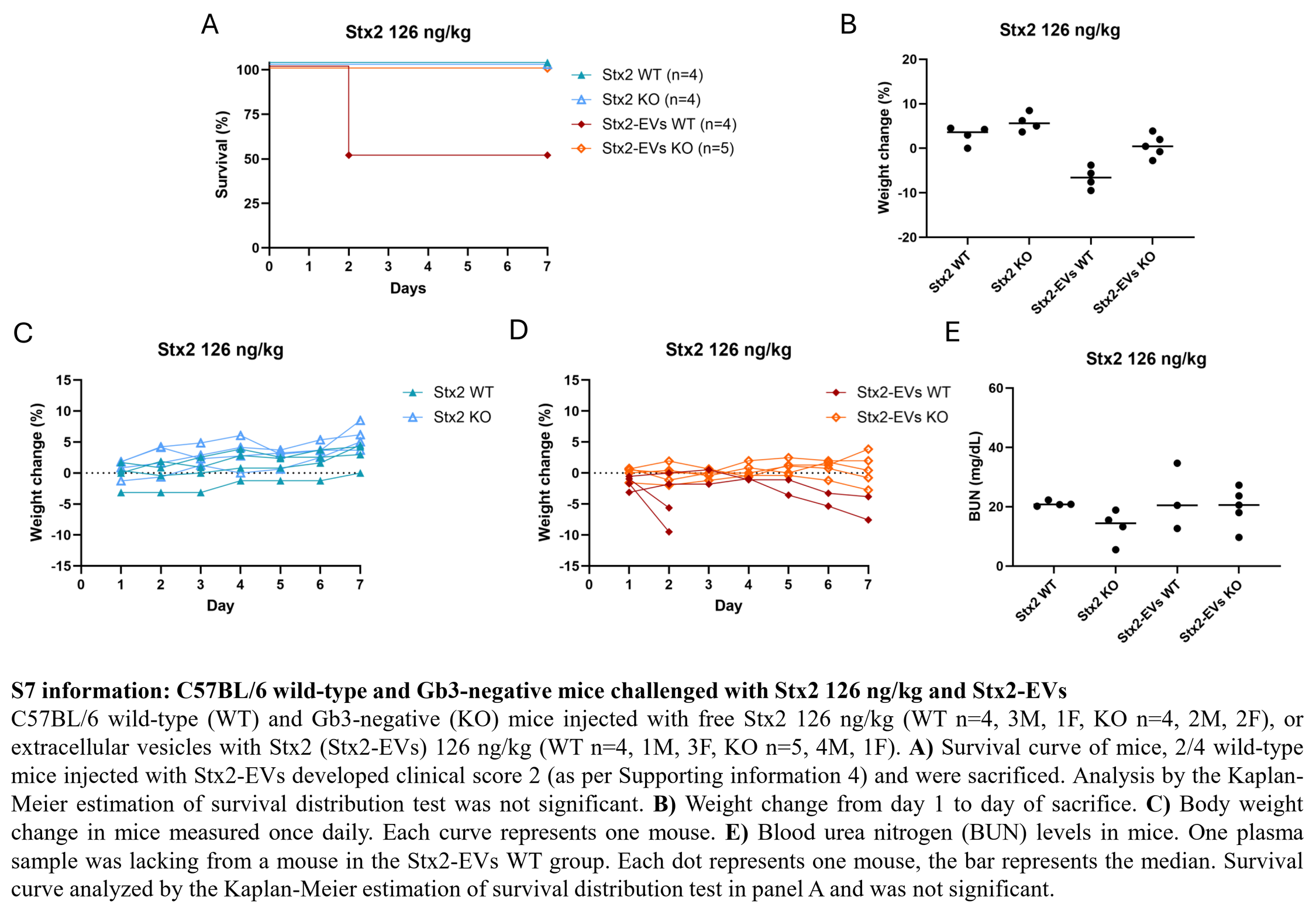

Supplement: S6 Fig — C57BL/6 wild-type (WT) and Gb3-negative (KO) mice injected with free Stx2 126 ng/kg (WT n = 4, 3M, 1F, KO n = 4, 2M, 2F), or extracellular vesicles with Stx2 (Stx2-EVs) 126 ng/kg (WT n = 4, 1M, 3F, KO n = 5, 4M, 1F). A) Survival curve of mice, 2/4 wild-type mice injected with Stx2-EVs developed clinical score 2 (as per S1 Table) and were sacrificed. Analysis by the Kaplan-Meier estimation of survival distribution test was not significant. B) Weight change from day 1 to day of sacrifice. C) Body weight change in mice measured once daily. Each curve represents one mouse. E) Blood urea nitrogen (BUN) levels in mice. One plasma sample was lacking from a mouse in the Stx2-EVs WT group. Each dot represents one mouse, the bar represents the median. Survival curve analyzed by the Kaplan-Meier estimation of survival distribution test in panel A and was not significant. (TIF) [file ppat.1014421.s007.tif]

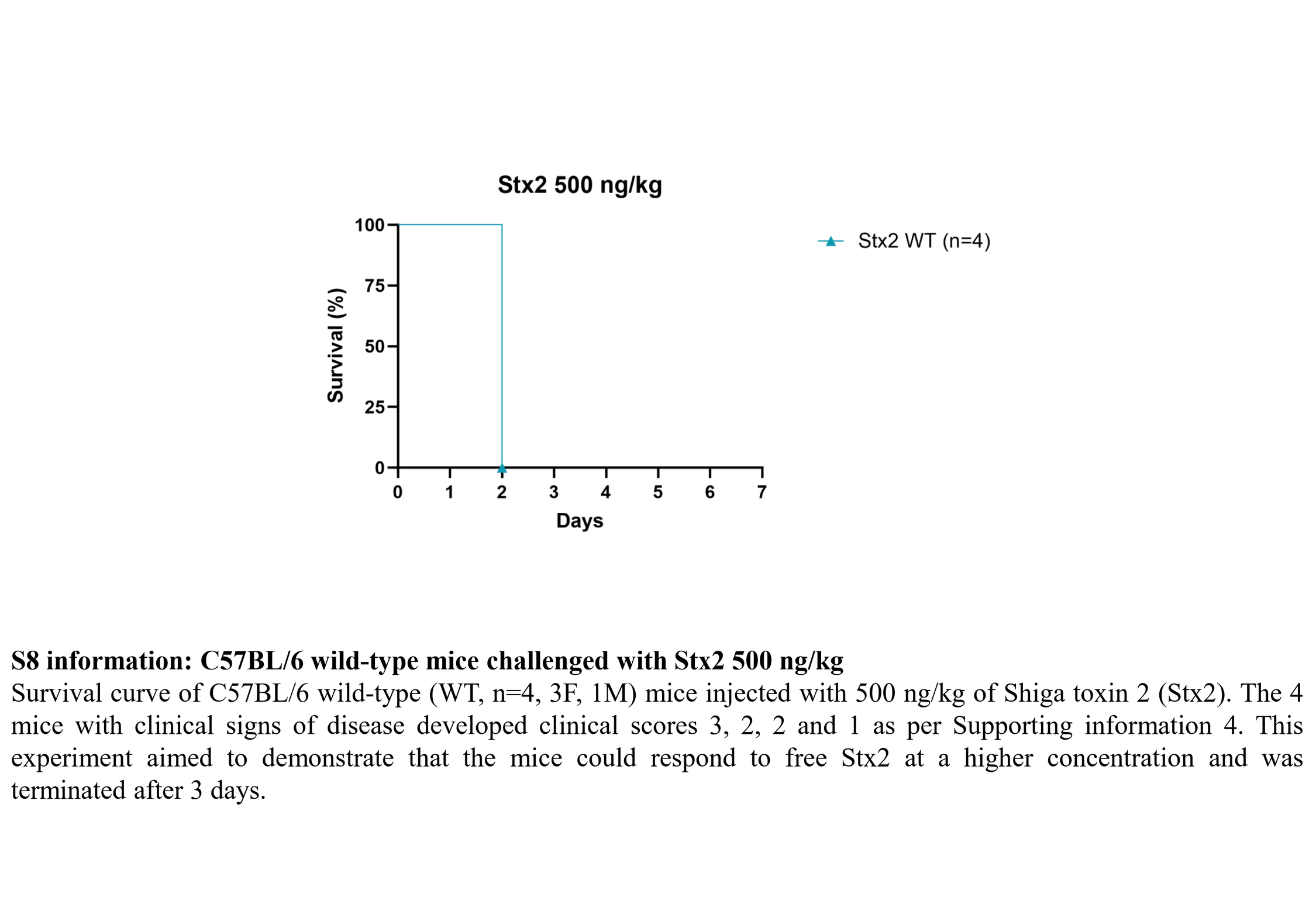

Supplement: S7 Fig — Survival curve of C57BL/6 wild-type (WT, n = 4, 3F, 1M) mice injected with 500 ng/kg of Shiga toxin 2 (Stx2). The 4 mice with clinical signs of disease developed clinical scores 3, 2, 2 and 1 as per S1 Table. This experiment aimed to demonstrate that the mice could respond to free Stx2 at a higher concentration and was terminated after 3 days. (TIF) [file ppat.1014421.s008.tif]

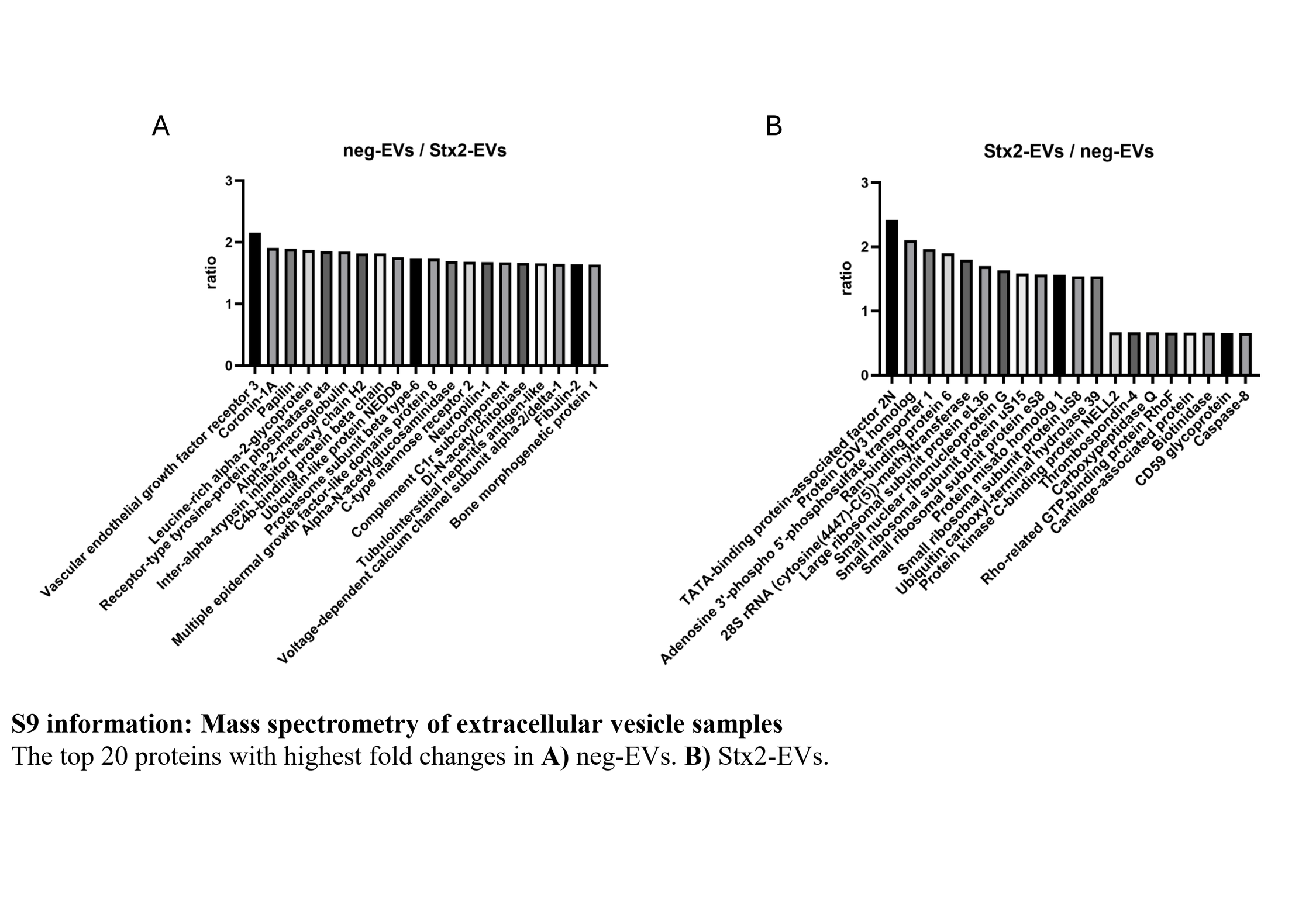

Supplement: S8 Fig — The top 20 proteins with highest fold changes in A) neg-EVs. B) Stx2-EVs. (TIF) [file ppat.1014421.s009.tif]

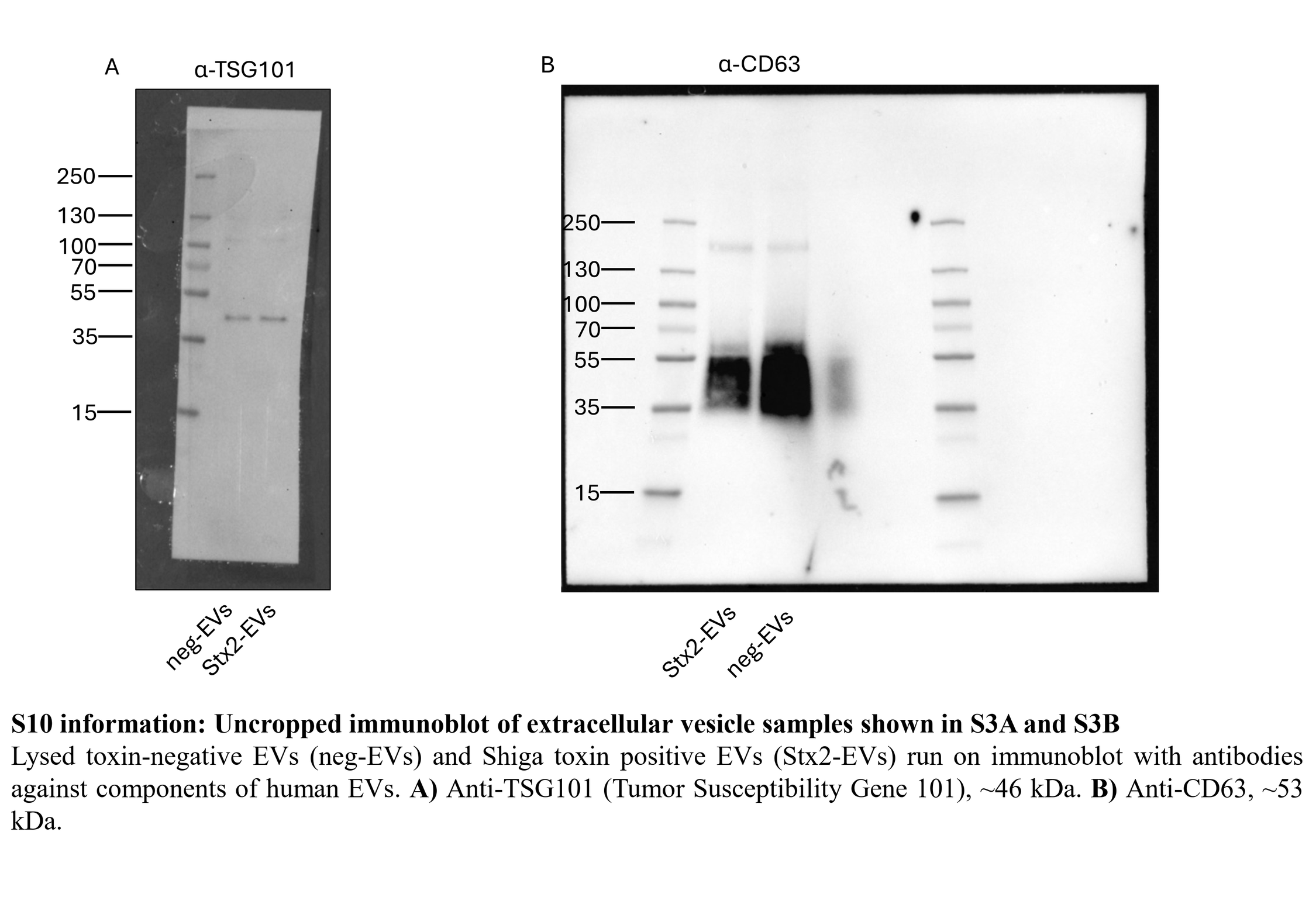

Supplement: S9 Fig — Lysed toxin-negative EVs (neg-EVs) and Shiga toxin positive EVs (Stx2-EVs) run on immunoblot with antibodies against components of human EVs. A) Anti-TSG101 (Tumor Susceptibility Gene 101), ~46 kDa. B) Anti-CD63, ~53 kDa. (TIF) [file ppat.1014421.s010.tif]

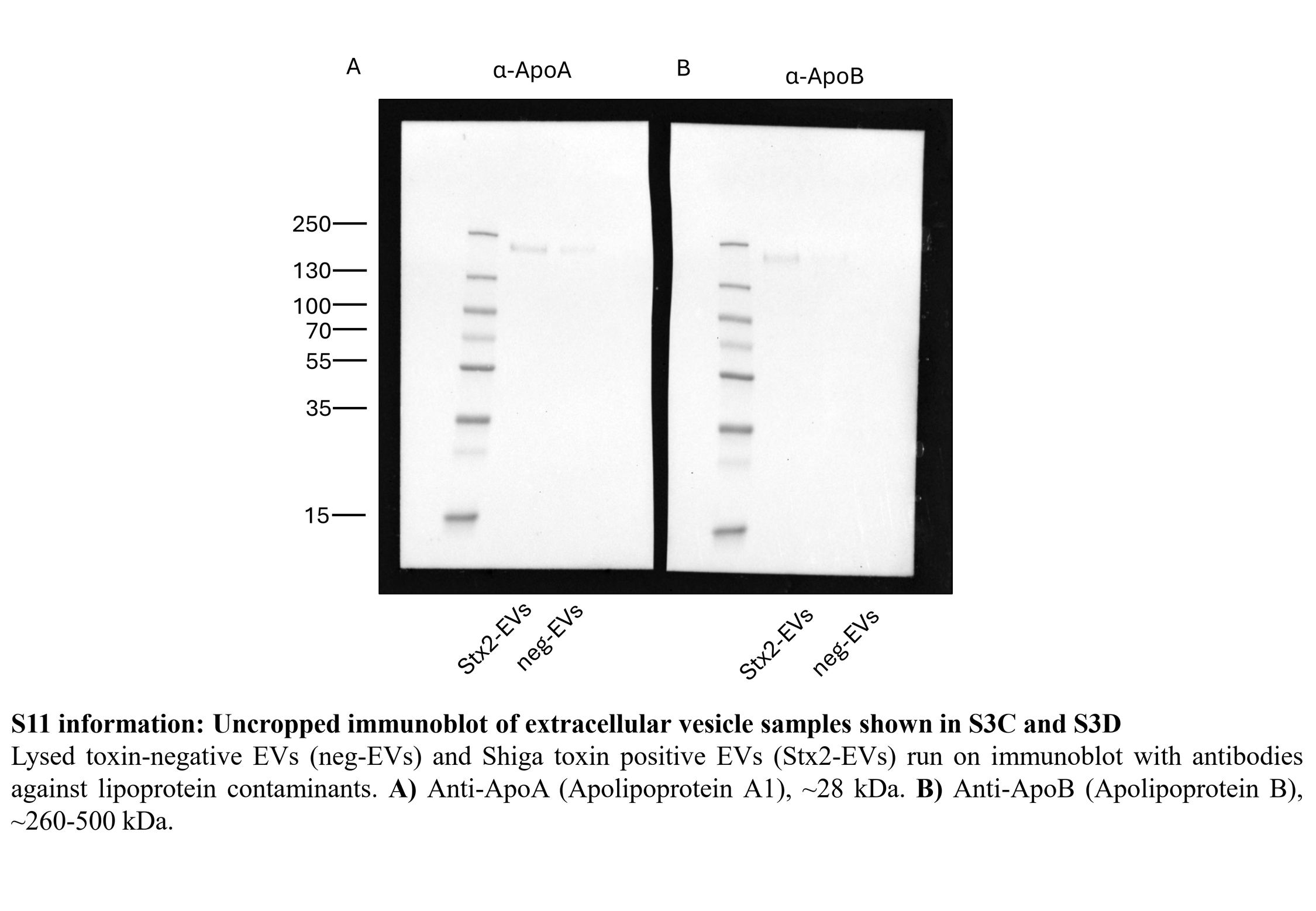

Supplement: S10 Fig — Lysed toxin-negative EVs (neg-EVs) and Shiga toxin positive EVs (Stx2-EVs) run on immunoblot with antibodies against lipoprotein contaminants. A) Anti-ApoA (Apolipoprotein A1), ~28 kDa. B) Anti-ApoB (Apolipoprotein B), ~260–500 kDa. (TIF) [file ppat.1014421.s011.tif]
